# Effects of home-based cardiac telerehabilitation programs in patients undergoing percutaneous coronary intervention: a systematic review and meta-analysis

**DOI:** 10.1186/s12872-023-03120-2

**Published:** 2023-02-22

**Authors:** Wen Zhong, Chenying Fu, Lin Xu, Xin Sun, Shiqi Wang, Chengqi He, Quan Wei

**Affiliations:** 1 Department of Rehabilitation Medicine Center and Institute of Rehabilitation Medicine, West China Hospital, Sichuan University, Chengdu, Sichuan People’s Republic of China; 2 Key Laboratory of Rehabilitation Medicine in Sichuan Province, Chengdu, Sichuan People’s Republic of China; 3 State Key Laboratory of Biotherapy and National Clinical Research Center for Geriatrics, West China Hospital, Sichuan University, Chengdu, Sichuan People’s Republic of China; 4 Aging and Geriatric Mechanism Laboratory, West China Hospital, Sichuan University, Chengdu, Sichuan People’s Republic of China

**Keywords:** Home-based cardiac telerehabilitation, Coronary artery disease, Percutaneous coronary intervention, Meta-analysis

## Abstract

**Background:**

Recent advances in telecommunications technology have raised the possibility of telehealth intervention delivering cardiac telerehabilitation, which may provide the efficacy of health services in patients after percutaneous coronary intervention (PCI). This study aimed to investigate the effects of home-based cardiac telerehabilitation (HBCTR) in patients undergoing PCI.

**Methods:**

We performed a comprehensive search of the following electronic databases: PubMed, Cochrane Central, Web of Science, Embase, CNKI, and WANFANG. For the prespecified outcomes, the primary outcomes were results of physical function (the six-minute walking test, 6MWT) and quality of life (QoL) of the participants. The secondary outcomes were results of (1) blood pressure; (2) full lipid profile (3) reliable assessment of anxiety and depression in patients.

**Results:**

All studies were conducted between 2013 and 2022, and a total of 5 articles could be included in the quantitative meta-analysis. The results showed that there was a statistically significant difference between the HBCTR intervention group and the control group in 6WMT (MD 16.59, 95%CI 7.13 to 26.06, *P* = 0.0006), but there was no difference in QoL (SMD  − 0.25, 95%CI  − 1.63 to 1.13, *P* = 0.73). According to the fixed effects model, there was a statistically significant difference between the HBCTR group versus the control group (MD − 2.88, 95%CI − 5.19 to − 0.57, *P* = 0.01), but not in diastolic blood pressure. Likewise, significant improvements of triglycerides and in low-density lipoprotein cholesterol were observed in HBTCR groups, but no significant differences were observed regarding total cholesterol and high-density lipoprotein cholesterol.

**Conclusion:**

This systematic review and meta-analysis have proven that the HBCTR is one of the promisingly effective cardiac rehabilitation strategies that improve cardiorespiratory fitness and reduce cardiovascular disease risk factors. With the continuous improvement of the telerehabilitation network, it is expected to serve in clinical.

## Introduction

Cardiovascular disease is the most predominant cause of death globally, with about estimated over 17 million people who died of cardiovascular disease in 2016, representing 31% of all deaths worldwide [1], among which coronary artery disease (CAD) remains one of the top killers [2]. CAD refers to the condition of vascular lumen stenosis or occlusion and vascular spasm based on coronary artery atherosclerosis, leading to myocardial ischemia, hypoxia, or necrosis [3]. CAD has become one of the causes of high morbidity and mortality and the leading cause of severe long-term disability in developed and some developing countries.

Percutaneous coronary intervention (PCI) is the primary way to obtain revascularization in patients with CAD [4] due to advances in PCI technology and technique [5]. After PCI, knowledge of cardiac rehabilitation (CR) and timely management of complications [6] are essential health services for patients, associated with decreasing the rate of vascular restenosis and recurrent ischemia, to improve quality of life [7]. As such, CR was recommended for secondary prevention, established by the American Heart Association and American College of Cardiology after PCI [4]. CR is a complete, full-cycle, and effective medical management strategy. However, the development of CR also faces many opportunities and challenges, such as the continuity of CR throughout the life cycle of patients [8, 9]. In the in-hospital rehabilitation period, the care team supervises the patient's daily life and motor ability to recover. But many patients do not transition to outpatient CR centers and receive recommended prescriptions in time after discharge [10]. Therefore, there are still gaps in this continuous medical behavior, which may eventually lead to unsatisfactory treatment effects and prognosis for patients. Despite the obvious evidence-based benefits, the participation rate of CR remains poor [11]. The reasons why people have low adherence to the traditional facility-based CR are multi-faceted [12], such as private insurance, the travel distance to a healthcare site and possibly affiliated CR facility, demographic and clinical factors, and existing comorbidity [13]. Therefore, it is reported that the center-based CR programs were challenged by low participant rates, insufficient attendance, and high drop-out rates. As a result, there is an urgent need for effective strategies to increase patient engagement, and home-based cardiac rehabilitation (HBCR) is one of the most potent strategies [14]. It also confirmed that the benefits of HBCR in terms of exercise capacity, control of risk factors, quality of life, and cost-effectiveness is similar to center-based CR [15, 16].But how to adequately assess the patient's situation and get timely feedback is also a major issue.

Recent advances in telecommunications technology have raised the possibility of telehealth interventions delivered by CR, which is able to overcome barriers of time and distance [17], and increase the rate of utilization mainly due to avoidance of expensive medical costs [18]. Therefore, we pay attention to the fact that home-based cardiac telerehabilitation (HBCTR) for patients in the home environment can link doctors and patients, better continue in-hospital rehabilitation, and also provide rehabilitation guarantee for out-hospital rehabilitation. Previous research has shown that the sooner CR begins in patients with CAD, the greater the benefit for patients [19]. CR for patients with CAD is divided into three stages, including stage I (in-hospital rehabilitation), stage II (out-of-hospital early rehabilitation or outpatient rehabilitation), and stage III (long-term community/family rehabilitation) [20]. Each stage of rehabilitation should follow the principle of safety. Therefore, most patients eligible for HBCTR are at low to intermediate risk, or in the transition from acute to convalescent phase and convalescent phase [14]. Telehealth can be defined as providing health management through emerging mobile devices such as mobile computing, medical sensor, and communications technologies [21]. The use of telehealth has grown tremendously and covers a wide range of content, such as digital information collection, precision medicine, virtual diagnosis, and treatment. Compared to other telehealth interventions, HBCTR focuses on the rehabilitation and prognosis of heart disease patients, and the core components of management include exercise training, risk factor control, psychological counseling, drug guidance, and nutritional prescription [22, 23]. The based model established by HBCTR is: the doctors formulate the CR prescription and send it remotely, and the patients execute the prescription, report data and conduct follow-up feedback, after that doctors make the personalized modification of the rehabilitation prescription in the standardized medical behavior. The closed-loop mechanism improves the patient's self-efficacy and enhances cardiac rehabilitation compliance. Meanwhile, HBCTR appears to be a more feasible and effective innovative rehabilitation model than conventional in-hospital CR [24]. Moreover, Stefanakis et al. showed patients received HBCTR with a low rate of adverse events after being fully evaluated before receiving the intervention [25].

It has been reported that telerehabilitation has proved beneficial effect for many patients, such as stroke survivors [26], patients with knee osteoarthritis [27], and patients after total knee arthroplasty [28, 29] and cardiovascular disease [30]. However, no systematic review of the effectiveness of HBCTR for patients after PCI has been substantially published. Therefore, this systematic review and meta-analysis aimed to investigate the benefits of telemedicine.

## Methods

This analysis was performed as following the Preferred Reporting Items for Systematic Review and Meta-Analyses (PRISMA) statement [31]. The review was registered in the International Prospective Register of Systematic Review (PROSPERO) Registry: CRD42021291148. All analyses involved were based on previously published studies. Thus, no ethical approval and patient consent were required.

### Literature search

We performed a comprehensive search of the following electronic databases for the potentially eligible studies until January 2023: PubMed, Cochrane Central, Web of Science, Embase, CNKI, and WANFANG. The keywords were performed in the following combinations: "percutaneous coronary intervention" AND "telemedicine or telerehabilitation or remote rehabilitation or e-health or telehealth or internet-based," with no time limit or language restrictions. A manual screening was performed for the reference lists of retrieved studies and relevant reviews to include additional eligible articles until no further report was identified.

### Study selection

The studies were included while they reached the specific criteria for this review were: (a) population: all participants hospitalized for documented coronary heart disease and treated with a successful PCI were included; (b) intervention: any form of the following technology conducted HBCTR, such as mobile phones, tablet computers, computers, television or video-conferencing; (c) control: the control group included usual care or active outpatient CR; (d)outcome: one of the following outcomes had to be reported: the six-minute walking test (6MWT), quality of life (QoL), blood pressure, total cholesterol (TC), triglycerides (TGs), low-density lipoprotein cholesterol (LDL-C), and high-density lipoprotein cholesterol (HDL-C), assessment of anxiety and depression; and (e) study design: randomized controlled trial design. We excluded articles that only focused on telerehabilitation systems development or patients assigned to HBCTR without systematic and regular rehabilitation treatment, which is just medical staff unilaterally reminding patients to pay attention to rehabilitation therapies by using social media such as emails, text messages, etc. And duplicate reports of the same team's study were not considered. Two reviewers first screened all titles and abstracts based on all searched results to identify all potentially relevant articles following the above criteria and then performed full-text filtering.

### Data extraction and outcomes

Two reviewers extracted data independently by using a developed Excel sheet. Information extracted from the relevant randomized controlled trials (RCTs) included: essential patient characteristics, data on sample size, study design, the intervention of telerehabilitation and control group, and outcome measures. For the outcomes, the primary outcomes were results of physical function (6MWT) and QoL of the participants. The secondary outcomes were results of (1) blood pressure, (2) full lipid profile (mmol/L) (3) reliable assessment of anxiety and depression in patients.

### Risk of bias

According to the Cochrane risk of bias tool, we conducted a quality assessment of the included studies by evaluating the risk of different forms of bias, such as selection bias, performance bias, detection bias, attrition bias, reporting bias, and other sources of bias. The PEDro scale also assessed the quality of the studies. All included trial reports were checked, and each item was rated as 'yes' or 'no.' Trials with higher scores are valid and reliable. If the judgments of two reviewers were uncertain, a third reviewer settled the discrepancy.

### Statistical analysis and outcome interpretation

All meta-analytic statistical analyses were performed with RevMan software for Windows (Version 5.3, The Cochrane Collaboration, Software Update, Oxford, UK) in this study. Mean differences (MDs) with 95% confidence intervals (CIs) for continuous outcome variables after therapy were used to estimate the total effects. The standardized mean difference (SMD) was calculated when studies used different scales to measure the same outcome, such as QoL. Statistical heterogeneity was assessed using the chi-square test, and *P* < 0.10 was considered statistically significant. The extent was measured using I^2^ tests, and I^2^ > 50% was regarded as high heterogeneity. Therefore, we used the random effects model meta-analysis when it had high heterogeneity and basically used a fixed effect model for meta-analysis.

## Results

### Study selection

**Fig. 1:**
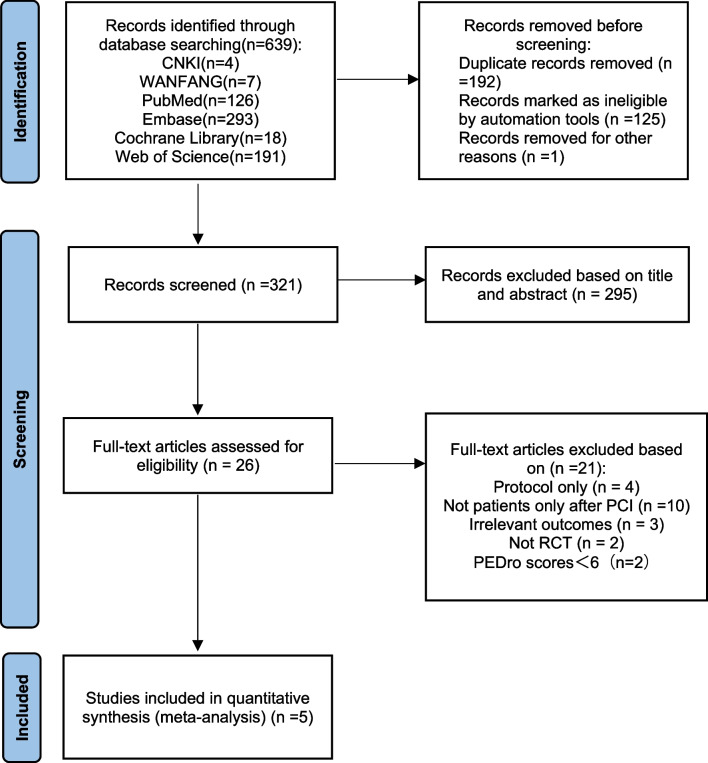
The PRISMA flow diagram for the study selection process

In all, we retrieved a total of 639 records to be potentially relevant through electronic searching from the six electronic databases, of which 192 duplicated studies and 126 ineligible studies marked by automation tools or for other reasons were removed. Then, 295 studies were excluded for not meeting the inclusion criteria. Only 26 records were identified as eligible after the initial screening of the title and abstract of 321 documents from 26 full-text papers screened. At the same time, only five articles fulfilled the absolute eligibility criteria, and all five articles [32–36] could be included in the quantitative meta-analysis. Figure 1 shows the details of our screening process.

### Study characteristics

The main forms of HBCTR are mobile device-based applications, wearables, and social media management platforms. The first form is to use applications to build HBCTR, mainly through remote assessment, electronic prescriptions, related health education, real-time doctor-patient online communication, and other ways. For example, Widmer et al. [36]and Dorje et al. [32] developed digital health interventions with smartphones as a carrier. The app provides patients with appropriate medical care and related information on CAD knowledge and awareness, as well as facilitates the monitoring and supervision of the physical activity. Patients also upload self-monitor information such as nutrition diaries, exercise records, and weight records through the software platform to guarantee the quality of HBTCR. The second form is the use of wearable devices such as heart rate belts and wristbands directly worn on the patient for CR, which can monitor heart rate and electrocardiogram. Wearable devices can not only urge patients to exercise, but also record exercise data, which is essential for the CR of patients with coronary heart disease. For example, Lee et al. [34] provided remote health education and exercise training for patients, who wore the provided wireless monitoring device (HeartCallTM, U-Heart, Korea) to monitor heart rate during exercise. At the same time, the researchers compared the patient's heart rate real-time heart rate with the target heart rate (target heart rate = (maximum heart rate—resting heart rate) × percentage + resting heart rate) and designed the patient's exercise intensity to increase from 40 to 80% in stages. Furthermore, Fang et al. [33]combined smartphones and wearable sensors used in HBCTR. This integrated telerehabilitation system has functions such as remote real-time exercise monitoring and guidance, and uses sensors to record the type of movement, duration of exercise, intensity, and frequency of daily activities of patients after being connected to a smartphone via Bluetooth, which is more accurate than self-reported physical activity. The third form is the formation of close doctor-patient connections through commonly used social media, with regular prompts and reminders, without the need for app development and algorithm optimization of wearable devices. For instance, Li et al. [35] used the WeChat platform, and they were divided into six groups according to the attending doctors, and 6 WeChat groups were established. One doctor and one nurse in each group followed the implementation of the exercise program. The baseline characteristics were summarized in Table 1.

**Table 1:** Characteristics of Included Studies

Author (Year) Country	Population			Intervention a. Telemonitoring and telecoaching design b. Exercise prescription (intensity, duration of exercise, modality of exercise)	Comparison	Outcome: a. Primary b. Secondary	Follow-up (months)
	Number(*n*)	Age (Mean ± SD)	Female, *n* (%)				
Widmer et al (2017) United States	IG: *n* = 37 CG: *n* = 34	IG: 62.5 ± 10.7 CG: 63.6 ± 10.9	IG: 8 (22%) CG: 5 (15%)	a. DHI + CR: patients receive the online and smartphone-based CR program and report their dietary and exercise habits to the DHI b. Standard phase II CR program (NA, 36 sessions/12 weeks, NA)	standard CR	a. CV-related ED visits and rehospitalizations b. Weight, blood pressure, heart rate, glucose/HbA1c, lipids, physical activity, diet, QoL, mood, compliance	3
Dorje et al (2019) China	IG: *n* = 156 CG: *n* = 156	IG: 59.1 ± 9.4 CG: 61.9 ± 8.7	IG: 28 (18%) CG: 19 (30%)	a. SMART-CR/SP: participants received educational modules by smartphone app delivered via WeChat, and patient-centered coronary heart disease risk factor monitoring and management support via WeChat-interfaced monitor b. The regular exercise protocol with daily step count supervision (NA, 1 per week/8 weeks and biweekly /24 weeks, NA)	Usual care	a. 6MWT b. Participants’ knowledge and awareness of coronary heart disease; resting heart rate; systolic blood pressure; cardiac rehabilitation and secondary prevention needs; full lipid profile; adherence to cardioprotective medications; smoking status; obesity (presented as BMI and waist-to-hip ratio); psychosocial wellbeing; and quality of life	12
Lee et al (2013) Korea	IG: *n* = 26 CG: *n* = 29	IG: 54.3 ± 8.9 CG: 57.8 ± 7.5	IG: 4(15%) CG: 7 (24%)	a. HBTCR: participants received a standard CR program, which consisted of educational rehabilitation and exercise training with wearing a wireless heart rate monitoring device during exercise b. The tailored exercise program (40% to 80% heart rate reserve, five times weekly/10 weeks, flexibility exercise for warm-up exercise for 10 min and cool-down exercise for 10 min, and main exercise of gait for 30 min)	Usual care	a. Exercise capacity (submaximal rate pressure product, submaximal rate of perceived exertion, metabolic equivalent of the tasks, and maximal exercise time other outcomes) b. Quality of life	3
Fang (2019) China	IG: *n* = 33 CG: *n* = 34	IG: 60.24 ± 9.35 CG: 61.41 ± 10.17	IG: 21(63.6%) CG: 21(61.8%)	a. HBCTR: participants received a real-time physiological monitoring system consisting of a belt strap with a sensor, a smartphone with an application, computer servers, and a web portal b. Individualized outdoor exercise (NA, no less than thrice per week/6 weeks, walking or jogging)	Usual care	a. 6MWT b. Clinical status (systolic blood pressure, diastolic blood pressure); anxiety and depression (CDS score); risk factors (FTND score); quality of life (SF-36 (PCS), SF-36 (MCS))	1.5
Li (2022) China	IG: *n* = 40 CG: *n* = 40	IG: 55.4 ± 8.9 CG: 55.6 ± 8.3	IG: 13(32.5%) CG: 15(37.5%)	a. HBCTR: received supervision intervention by a medical rehabilitation team, divided into six WeChat groups according to the doctor in charge, and they used an exercise bracelet to monitor heart rate b. The walking program (NA, NA, NA)	Outpatient rehabilitation treatment	a. Compliance, satisfaction evaluation, incidence of cardiac events b. Heart rate, quality of life score, and 6-min walking test	6

*IG* Intervention group; *CG* control group; *DHI* Digital health interventions; *CR* cardiac rehabilitation; *NA* Not Available; *CV* cardiovascular; *ED* emergency department; *QoL* quality of life; *SMART-CR/SP* a system involved smartphone-based home cardiac rehabilitation and secondary prevention program; *6MWT* the six-minute walking test; *BMI* body mass index; *HBTCR* home-based cardiac telerehabilitation; *CDS* the Cardiac Depression Scale; *FTND* the Fagerstrom Test for Nicotine Dependence questionnaire; *SF-36 (MCS)* SF-36 Health Survey (mental component summary scale); *SF-36 (PCS)* SF-36 Health Survey (physical component summary scale)

### Risk of bias assessment results

**Fig. 2:**
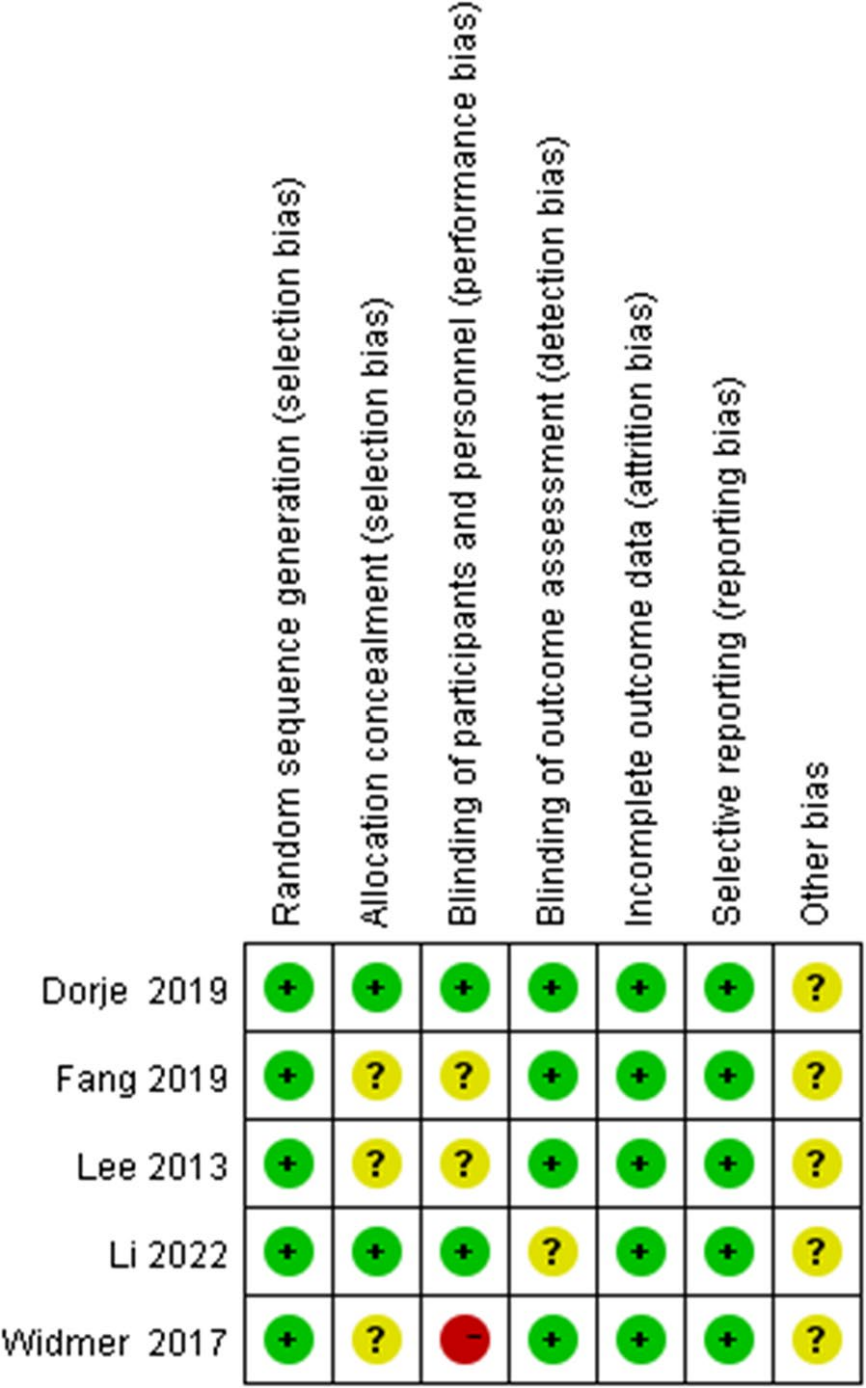
Risk of bias assessment summary according to the Cochrane risk of bias tool: red, green, and yellow colors indicate high, low, and unclear risk of bias, respectively

Quality assessment of the risk of bias was undertaken for included studies conducted by two authors independently. Since the participants could not be blinded to allocation due to the intervention's characteristics, there were high risks for performance bias in all included trials. The other risk of bias assessments of the included studies is summarized in Fig. 2. Furthermore, all five studies were assessed by the PEDro scale (Table 2) to evaluate the methodological quality of the included literature. Among them did not have inadequate blinding of participants and therapists, three studies did not reveal concealed allocation, and four did not perform an intention-to-treat analysis.

**Table 2:** The PEDro quality assessment of the included studies. Eligibility criteria did not contribute to the total score: 1 = yes (reported in study), 0 = no (not met)

Quality metric	Widmer et al.	Dorje et al.	Lee et al.	Fang et al.	Li et al.
Eligibility criteria	Yes	Yes	Yes	Yes	Yes
Random allocation	Yes	Yes	Yes	Yes	Yes
Concealed allocation	No	Yes	No	No	Yes
Baseline comparability	Yes	Yes	Yes	Yes	Yes
Blinded subjects	No	No	No	No	No
Blinded therapists	No	No	No	No	No
Blinded assessors	Yes	Yes	Yes	Yes	Yes
Adequate follow up	Yes	Yes	Yes	Yes	Yes
Intention-to-treat analysis	No	Yes	No	No	No
Between-group comparisons	Yes	Yes	Yes	Yes	Yes
Point estimates and variability	Yes	Yes	Yes	Yes	Yes
Total score	6	8	6	6	7

### Assessment of outcomes

#### Physical function

**Fig. 3:**
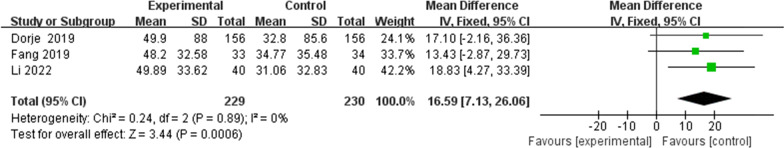
The six-minute walking test (6MWT)

Three studies [32, 33, 35] were included in the review to evaluate the change in the 6MWT. According to the fixed-effect model, there was statistically a significant difference between two groups (MD 16.59, 95%CI 7.13 to 26.06, *P* = 0.0006) with no heterogeneity among these studies (*P* = 0.89, I^2^ = 0%) (Fig. 3). Although Lee et al. did not include the 6MWT, studies have shown a significant increase in metabolic equivalent of the tasks (METs) (+ 34.6%) in the HBCTR group.

#### QoL

**Fig. 4:**
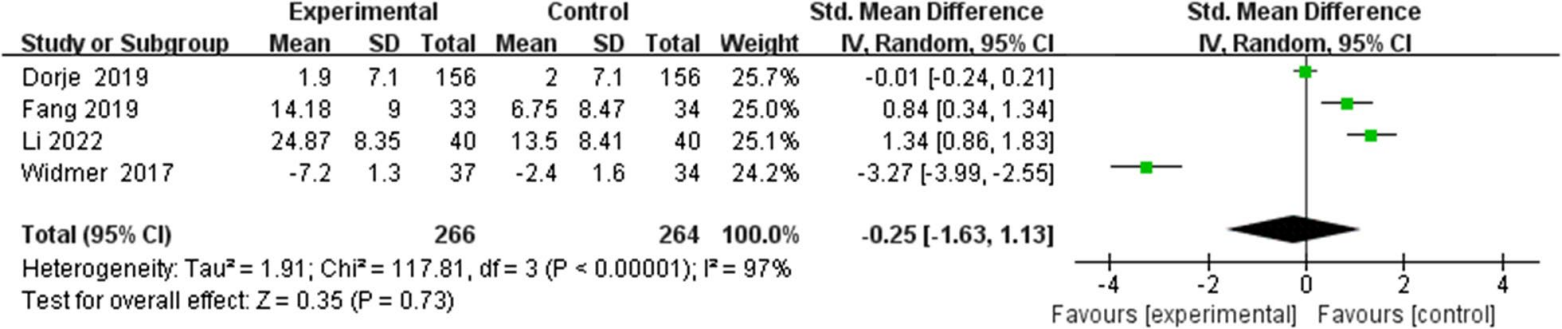
Quality of Life (QoL)

Four studies [32, 33, 35, 36] reported on the QoL of patients. Due to the different scales used for measurements, such as Dartmouth quality of life, the 12-item short form health survey (SF-12) and the 36-item short form health survey (SF-36), the standardized mean difference was used as the effective index. However, there was no significant difference between the HBCTR group versus the control group (SMD  − 0.25, 95%CI  − 1.63 to 1.13, *P* = 0.73) with statistical heterogeneity among these studies (*P* < 0.00001, I^2^ = 97%) (Fig. 4).

#### Blood pressure

**Fig. 5:**
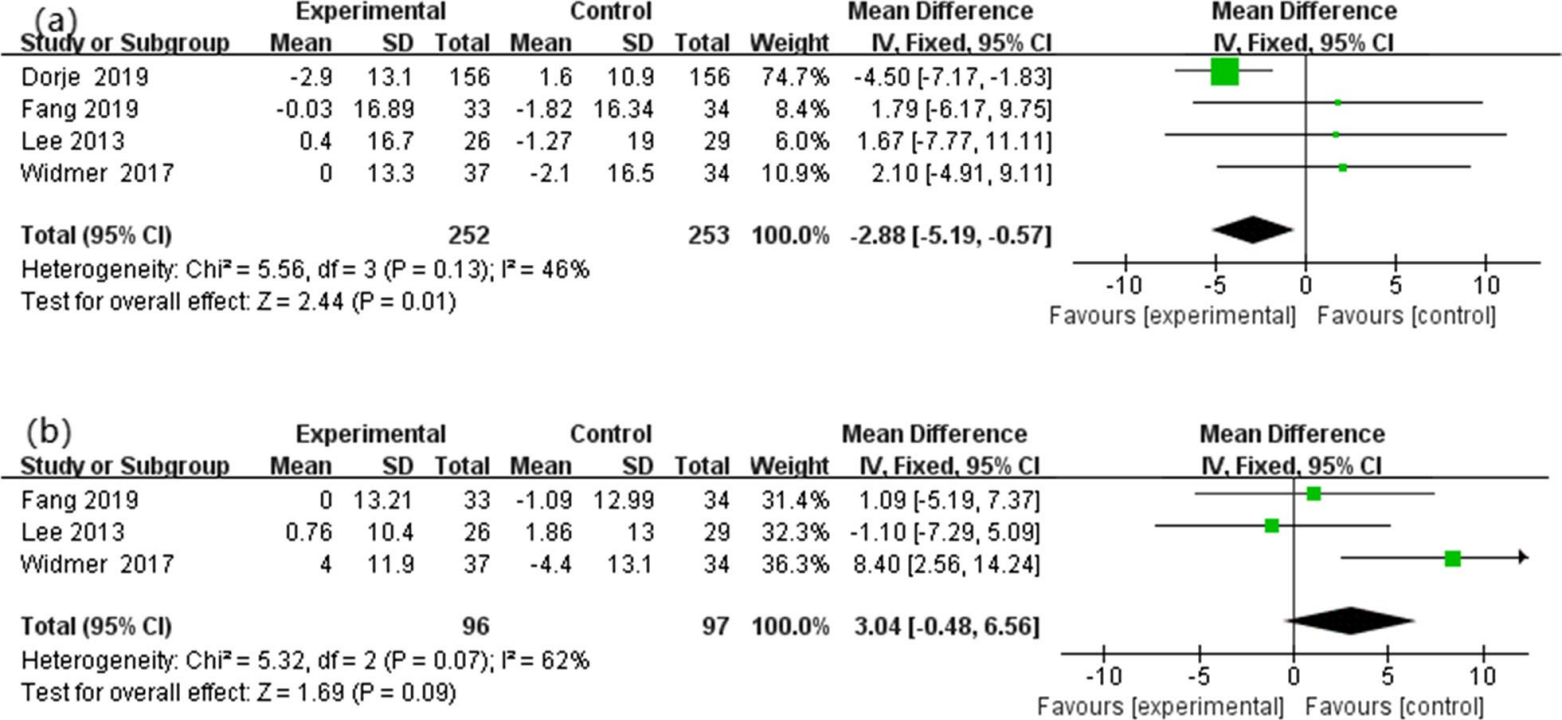
Blood pressure: **a** systolic blood pressure and **b** diastolic blood pressure

Four studies [32–34, 36] were included in the review to evaluate the change in systolic blood pressure. According fixed effects model, there was statistically significant difference between the HBCTR group versus the control group (MD  − 2.88, 95%CI  − 5.19 to  − 0.57, *P* = 0.01) with no high heterogeneity among these studies (*P* = 0.13, I^2^ = 46%). Nevertheless, only three articles [33, 34, 36] have reported on diastolic blood pressure. According to the fixed-effect model, there was no significant difference between the two groups (MD 3.04, 95%CI  − 0.48 to 6.56, *P* = 0.09) (Fig. 5).

#### Blood lipid concentrations

**Fig. 6:**
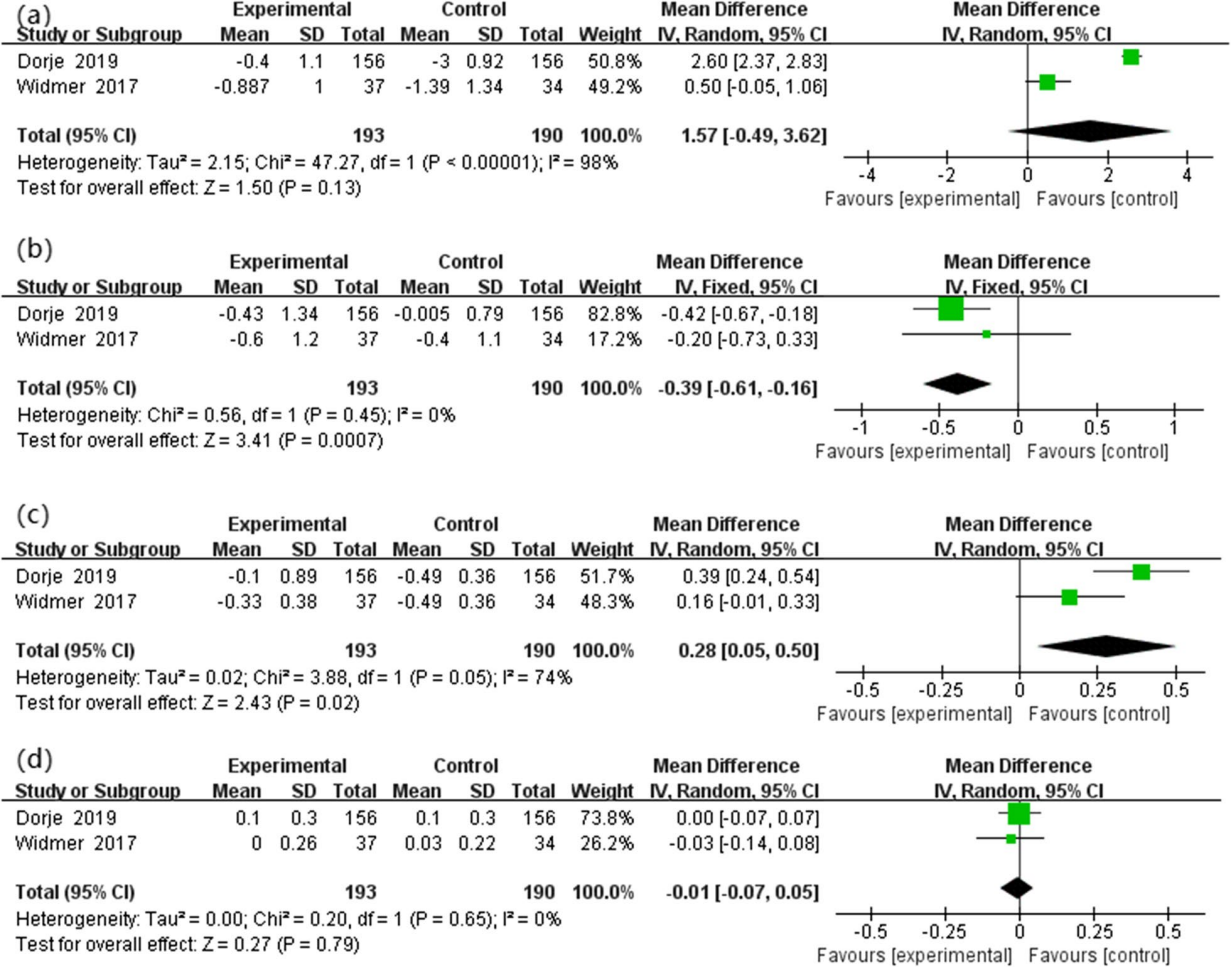
Blood lipid concentrations: **a** the change of total cholesterol (TC), **b** triglyceride (TG), **c** low-density lipoprotein cholesterol (LDL-C) and **d** high-density lipoprotein cholesterol (HDL-C)

Two studies reported data on blood lipid concentrations. All were included in the review to evaluate the change in TC, TGs, LDL-C and HDL-C. According to the fixed effects model, there was a statistically significant difference between the two groups in TG (MD  − 0.39, 95%CI  − 0.61 to  − 0.16, *P* = 0.0007). Meta-analysis of the included trials also shows significant differences in LDL-C (MD 0.28, 95%CI 0.05 to 0.50, *P* = 0.02). However, there was no statistically significant difference in TC and (MD 1.57, 95%CI  − 0.49 to 3.62, *P* = 0.13) and HDL-C (MD  − 0.01, 95%CI  − 0.07 to 0.05, *P* = 0.79) (Fig. 6).

## Discussion

This systematic review and meta-analysis explored the effects of telehealth interventions delivered to cardiac telerehabilitation patients after PCI. The primary outcomes we focus on are exercise capacity and QoL. The findings of this meta-analysis show that patients undergoing HBRCT can significantly improve physical exercise capacity (6WMT) but do not observe a statistically significant improvement in QoL. Secondary outcomes focused on blood pressure and lipids, and HBCTR significantly improved systolic blood pressure but not diastolic blood pressure. Similarly, there were statistically significant differences in TGs and LDL-C between the two groups but no significant differences in TC and HDL-C.

Exercise capacity is a vital measure of the effectiveness of CR in patients with coronary heart disease and is associated with all-cause and cardiovascular mortality. There is also evidence that increased physical activity, exercise training, and overall cardiorespiratory fitness are protective in preventing coronary heart disease [37]. The Peak oxygen consumption (VO_2_ peak) in the cardiopulmonary exercise test (CPET) is the gold standard for reflecting the patient's cardiorespiratory fitness and exercise capacity [38]. During the test, the patient was set up with a gradual increase in load power. When the patient exercises to a certain extent, cell uptake of oxygen appears a plateau, that is, even if the test power is increased, oxygen uptake does not increase, which is VO_2_ peak at this time. A recent systematic review and meta-analysis [39] showed that HBTCR assisted by wearable sensors improved cardiorespiratory fitness in people with CVD. Similarly, Duscha et al. [40] showed that the HBCTR sustain the gains in VO_2_ peak compared to the usual care group. In addition, indicators such as 6MWT, the daily activity time, and daily walking steps can also reflect the patient's cardiorespiratory fitness. Because finally included article only reflected the results of the 6MWT and did not measure by CPET, our meta-analysis focused on 6MWT as the primary outcome. The 6MWT results lack accuracy compared to VO_2_ peak, but it is also the basis for prescribing exercise for patients as a submaximal exercise capacity test. For the determination of exercise intensity, as described in the study by Luo et al. [41], the average velocity of 6MWT correlated well with the anaerobic threshold of CPET. It is an easy and efficient way to correlate closely with METs values at the anaerobic threshold [42]. At the same time, the 6MWT can be used to judge the degree of disease progression in patients with heart disease, and it also has prognostic value related to cardiorespiratory health.

In our results, telerehabilitation improved 6WMT, which is consistent with previous studies. Schopfer et al. [43] found that patients allocated in-home rehabilitation group achieved a more remarkable 3-month improvement in 6MWT distance (+ 95 vs. + 41 m; *P* < 0.001). Moreover, a recent meta-analysis [44] showed a significant improvement in the 6-min walk test distance of functional ability participating in HBCTR in 14 randomized controlled trials. HBCTR improves exercise capacity and cardiorespiratory fitness primarily through exercise prescription. Exercise training CR can reduce oxidative stress, improve endothelial progenitor cell function, improve ventricular remodeling and regulate inflammation [45]. These mechanisms dilate the coronary arteries and establish collateral circulation, as mentioned in the study by Mj et al. [46], and exercise also increases blood flow and myocardial energy supply by enlarging the luminal area of collateral vessels and increasing myocardial capillary density [47]. Therefore, telerehabilitation can improve the cardiopulmonary function of patients, thereby increasing the patient's exercise ability and physical activity, which is beneficial to the QoL (MD 25.58 m, 95%CI 14.74 to 36.42).

Another interesting finding of our review is that the improvement of HBCTR in QoL was comparable to that of the control group, with no statistically significant difference. Although our results show that telerehabilitation improves patients' physical capacity, it is closely related to patients' QoL after PCI, including physical and psychological functions. And anxiety and depression in patients with coronary heart disease will seriously affect the QoL [48]. Therefore, we performed a meta-analysis of anxiety or depression scores in the included articles. The research results of included articles show that telerehabilitation training has no obvious advantages in improving negative anxiety and depression (anxiety: MD  − 0.03, 95%CI  − 0.16 to 0.21, *P* = 0.0006; depression: MD  − 0.43, 95%CI  − 1.41 to 0.55, *P* = 0.0006). The reason for this may be that fewer studies were included and that the duration or intensity of the exercise intervention was insufficient to detect the effect of the intervention. Therefore, confirming the value of telemedicine in this regard should be the focus of future research.

At the same time, some modifiable risk factors, such as blood pressure and blood lipid level, were also focused on in our review. Blood pressure management for Patients Undergoing PCI should be aimed at reducing cardiovascular events [49]. Our findings show that telerehabilitation can effectively improve systolic blood pressure, which may be enhanced by exercise and lifestyle. It reduces sympathetic nerve activity [50], antagonizes the renin–angiotensin–aldosterone system [51], attenuates inflammatory responses and oxidative stress, and improves endothelial dysfunction and vascular remodeling [52]. Then, our results showed that telerehabilitation did not significantly improve diastolic blood pressure in patients, which may be related to the fluctuation of blood pressure for patients after PCI [53]. Studies have shown that the blood vessel wall cannot be significantly contracted or relaxed in patients with coronary heart disease due to atherosclerosis, which may lead to high diastolic blood pressure and difficulty controlling [54]. Therefore, further research is needed to improve the diastolic blood pressure of patients with coronary heart disease by telerehabilitation. In terms of blood lipids, the results showed that HBCTR improved TGs and LDL-C, with no statistically significant improvement in TC and HDL-C. Similar to previous findings, such as in the randomized controlled trial of Pfaeffli et al. [55], patients with CAD in the experimental group received 6 months of personalized telehealth intervention with online support and found that the LDL-C levels of the experimental group were lower than those in the control group. In the trial of Dalli-Peydró et al. [56], who used a smartphone app to instruct participants through exercise schedules and communicate with patients via text message, the results showed that prevented deterioration of the TG/HDL ratio. And one study [57] has shown that if patients with acute myocardial infarction who received PCI and standard medical therapy after 1 year follow-up of LDL-C still ≥ 1.8 mmol/L, more than 65% of patients had an increased risk of long-term death by 42% ~ 45%. Therefore, HBCTR is crucial for the control of risk factors such as blood lipids, which can not only supervise the standardized use of lipid-lowering drugs in patients after PCI surgery, but also further control blood lipids to the ideal level through exercise and lifestyle improvement.

PCI can quickly restore the blood circulation of coronary arteries, improve myocardial ischemia and save heart function. It's been mentioned in many articles that patients after PCI need to pay attention to improving exercise capacity, lower cardiovascular risk profile, and increasing physical functioning, which is associated with an increased incidence of late cardiovascular events [7, 58]. So, CR is a valuable treatment for patients After PCI [59]. Patients after PCI in CR are associated with improved quality of life, reduced readmission rates, and cardiovascular mortality. Hospital-based or center-based CR programs were reportedly challenged by low participant uptake, insufficient attendance, and high drop-out rates. And under telehealth intervention, the benefits are significantly greater because it is more convenient, flexible, and easier to access [60]. Telehealth intervention delivered cardiac telerehabilitation is defined as a telemedicine program that implements telemedicine rehabilitation services for patients by using remote leading media technology. HBCTR includes many aspects, such as remote follow-up, exercise training, health education guidance, remote treatment, monitoring, etc. It mainly sends personalized rehabilitation programs to patients through the network system to improve their conditions for rehabilitation, enhance patients' compliance and persistence in rehabilitation, and save time and cost. At the same time, the patient's self-monitoring and self-evaluation are also crucial for HBCTR. Karen et al. [61] found in the study of 172 patients with acute myocardial infarction that the HBCTR group significantly improved the patient's self-management ability, which was also confirmed by Maddison et al. [62]. And in a descriptive qualitative study of 20 patients in the intervention group using the eHealth CR website, the results showed that telerehabilitation provides social support for changes in patients' cognitive determinants during the intervention [63].

As a result, our goal was to investigate the effect of the HBCTR for patients undergoing PCI. These results are comparable with the results of other meta-analyses. Clark et al. [17] and Neubeck et al. [64] showed that significant favorable changes in TC and systolic blood pressure with telehealth interventions were observed in a meta-analysis. Avila et al. [65] also showed that exercise capacity remained stable during one year following phase II cardiac rehabilitation by home-based exercise with telemonitoring guidance but also using wrist HR monitors. In Batalik et al.'s study [66], each patient received feedback, motivation, and education through telehealth, and the researchers used the Global positioning system to supervise the patient's training site. Helping to self-regulate lifestyle through motivational coaching strategies and objective feedback on training data is an important part of cardiac rehabilitation execution. And the primary findings include evidence that HBTCR is more effective than center-based CR at maintaining long-term cardiorespiratory fitness levels. However, our review is not exactly consistent with previously published systematic reviews, like the FIT@Home study [67], a heart rate monitor with a chest strap and a web application uploaded recorded heart rate data via the Internet were used to guide the exercise process of home telerehabilitation. However, the results showed that there was no significant difference in physical fitness between home exercise training and central exercise training guided by remote monitoring. And our systematic review has some new strengths. We investigated the participants who were restricted after PCI. Post-PCI patients urgently need self-management to improve clinical outcomes, such as reducing depression and anxiety, reducing mortality and morbidity, and improving health-related quality of life (HRQoL) [68]. Compared with a former systematic review, exercise training is a core component in previous studies, but we performed this including some multidisciplinary interventions and multifaceted care, such as physical exercise, nutritional advice, and target-driven pharmacological therapies.

## Limitations

There are some limitations to this study. The first limitation is the great variability and complexity of intervention models, such as different frequency and intensity forms. Moreover, the included studies used various models of telerehabilitation (different duration, frequency, length, and intensity). For example, there were a wide of telehealth intervention models, such as smartphone-based CR platforms, remote monitoring systems, wireless monitoring, and sports band with a smartphone. Therefore, future research needs to explore which model is best for these patients. Second, some results could not be quantitatively analyzed due to the relatively small sample size of the included studies. Third, we only focused on treatment efficacy and need to pay attention to operability and cost of services, which should be included in future studies. Therefore, more extensive randomized controlled trials are required in order to confirm the current evidence.

## Conclusions

This systematic review and meta-analysis have proved that HBCTR can effectively improve patients' physical function after PCI. These results justify that the home-based telehealth intervention is one of the promisingly effective CR strategies that reduce cardiovascular disease risk factors. In order to further confirm HBCTR increasing uptake and make CR available, the sample size needs to be increased, and future research needs to explore which model is best for these patients.

## Data Availability

All data generated or analysed during this study are included in this published article.

## Abbreviations

CAD: Coronary artery disease
PCI: Percutaneous coronary intervention
CR: Cardiac rehabilitation
HBCR: Home-based cardiac rehabilitation
HBCTR: Home-based cardiac telerehabilitation
6MWT: The six-minute walking test
QoL: Quality of life
TC: Total cholesterol
TGs: Triglycerides
LDL-C: Low-density lipoprotein cholesterol
HDL-C: High-density lipoprotein cholesterol
RCTs: Relevant randomized controlled trials
VO_2_ peak: Peak oxygen consumption
CPET: Cardiopulmonary exercise test
